# Treatment Satisfaction With Couplelinks Online Intervention to Promote Dyadic Coping in Young Couples Affected by Breast Cancer

**DOI:** 10.3389/fpsyg.2022.862555

**Published:** 2022-06-16

**Authors:** Karen Fergus, Adina Tanen, Saunia Ahmad, Sandra Gardner, Ellen Warner, Deborah McLeod, Joanne Stephen, Wendy Carter, Amanda Periera

**Affiliations:** ^1^Psychosocial Oncology Lab, Department of Psychology, York University, Toronto, ON, Canada; ^2^Odette Cancer Centre, Sunnybrook Health Sciences Centre, Toronto, ON, Canada; ^3^School of Medicine, University of Toronto, Toronto, ON, Canada; ^4^Toronto Psychology Clinic, Toronto, ON, Canada; ^5^Rotman Research Institute, Baycrest Health Sciences, Toronto, ON, Canada; ^6^Division of Biostatistics, Dalla Lana School of Public Health, University of Toronto, Toronto, ON, Canada; ^7^School of Nursing, Dalhousie University, Halifax, NS, Canada; ^8^Clinical Neurosciences, Alberta Health Services, Calgary, AB, Canada; ^9^Toronto Academic Pain Institute, Women’s College Hospital, Toronto, ON, Canada

**Keywords:** breast cancer, couples, intervention, online, dyadic coping, psychosocial, satisfaction, young

## Abstract

**Background:**

This study evaluated participant satisfaction with “Couplelinks,” an online psychological intervention designed for younger couples coping with breast cancer. The program included six experiential learning exercises (plus one optional module), psychoeducational information, and support from a personal mental health professional.

**Objective:**

The primary objectives were to examine participants’ perceptions of: the online intervention’s structure and content; the value of including a professional facilitator; and benefits and drawbacks of the program.

**Methods:**

A treatment satisfaction questionnaire comprised of Likert indices and open-ended questions pertaining to treatment satisfaction was completed by 26 patients and 27 male partners (*N* = 53) approximately 1–2 weeks following the intervention which occurred in the context of a randomized controlled trial. Descriptive statistics were used to summarize satisfaction ratings and generalized linear models with fixed effect for gender were used to test for differences in male-female outcomes. A thematic analysis was undertaken in order to understand, organize and summarize the qualitative textual feedback.

**Results:**

Participants reported an overall satisfaction rating of 4.3 out of 5 (*SD* = 0.54) with patient satisfaction ratings being higher than that of male partners’ (*p* = 0.01). The majority of participants considered the facilitator’s role to be necessary 4.6 (*SD* = 0.60), and found the program to be convenient 4.1 (*SD* = 0.81) despite some participants struggling to keep up with the modules. Subjective data revealed participants valued the convenience and flexibility of the online intervention and appreciated the program’s involvement of both partners. Participants also reported that including a professional facilitator humanized the intervention, served as motivation to progress through the program, facilitated insight into their relationship, and was reassuring. Experiential gains noted by participants included that the program: helped couples to open channels of communication; prompted them to designate quality time for one another; evoked feelings of unity and togetherness; and inspired new insight in the relationship.

**Conclusion:**

Such feedback supports the feasibility and acceptability of the Couplelinks program while offering directions for improvement of online couple-based interventions in cancer.

## Introduction

Women diagnosed with breast cancer (BC) during their childbearing years tend to face challenges that make accessing traditional psychosocial supports particularly burdensome because they are often juggling myriad family and employment-related responsibilities alongside invasive and taxing treatments (Gould et al., 2006). The quality of life and psychological wellbeing of women with BC under age 50 tend to be poorer than that of older woman (Reis, 2007), ascribable to the unique frustrations and challenges they face because of their younger age (Ali and Warner, 2013; Acquati and Kayser, 2019). Moreover, BC diagnosed early in life tends to be more aggressive and have a worse prognosis (Stamatakos et al., 2011), and yet, young women report having more difficulty accessing relevant information about BC than do older women (Gould et al., 2006). In one large-scale longitudinal study of women who were diagnosed with BC prior to the age of 50 and assessed at 5- and 10-years post-diagnosis, BC survivors reported diminished quality of life including reduced physical wellbeing and sexual activity (Bloom et al., 2012)—a finding consistent with other studies of younger couples and BC (Walsh et al., 2005; Fiszer et al., 2014).

Although it is the woman who is directly affected by the life-threatening diagnosis and burden of treatment, the challenges and psychological distress associated with the illness also extend to her partner. Male partners of patients with BC tend to experience reduced psychological wellbeing, lower quality of life, and lower sexual engagement compared to pre-diagnosis (Fletcher et al., 2010; Badr and Krebs, 2013). They are also often preoccupied by many anxieties such as the cancer’s potential return, the emotional wellbeing of their children, how to behave in a supportive role, and how to be helpful in a practical way (Fletcher et al., 2010). As well, male partners often neglect their own self-care by putting their own needs on hold while supporting their wives’ needs during cancer treatment, and keeping home life in order (Hilton et al., 2000).

BC also generates challenges that are psychologically distressing to both partners concurrently. For example, due to the more common need for gonadotoxic chemotherapy when treating younger BC patients, potential infertility is a distressing fear for many young couples who have not started or completed their families (Stamatakos et al., 2011). Sexual dysfunction post BC treatment is another source of anxiety and tension within the relationship (Hilton et al., 2000; Stamatakos et al., 2011). Given the numerous challenges faced by younger couples undergoing cancer treatment or coping with its after-effects, support programs specifically designed to address their psychological needs as individuals and an intimate dyad are essential.

### Dyadic Coping and Adjustment to Cancer

Dyadic coping ability is associated with improved couple adjustment to BC and reductions in the individual psychological distress of each partner (Berg and Upchurch, 2007; Heinrichs et al., 2012; Kayser et al., 2017). Couples who employ dyadic coping strategies demonstrate greater reductions in BC-related fears, less avoidance in dealing with the cancer, and more posttraumatic growth (Heinrichs et al., 2012). Greater levels of “we-ness” are also positively associated with a woman’s confidence in dealing with BC-related stressors, contributing to an easier adjustment to cancer (Ahmad et al., 2017). Research also demonstrates that the more dyadic coping skills couples utilize, the greater reductions in psychological distress the BC patient and her partner will experience (Rottmann et al., 2015). Improving relationship quality and facilitating feelings of support and intimacy through dyadic coping is therefore an important goal of interventions for couples affected by BC.

### Online Interventions for Couples

The development and evaluation of online interventions as a flexible and accommodative alternative to traditional, in-person therapy in cancer care has been a burgeoning area of research. Further support for the feasibility of online modalities in cancer care comes from a review by Yoon (2013) that found online interventions to yield a high rate of satisfaction among cancer patients and their caregivers. According to Yoon, the perceived benefits of online interventions included the flexibility of using the intervention on one’s own time, its ability to foster communication about delicate, cancer-related topics, and its efficient transfer of scientifically vetted information.

In regard to couples in particular, Doss et al. (2016) successfully translated an in-person therapy program for heterosexual couples into an 8-h online program called OurRelationship.com. The participating couples in this large-scale study reported significant improvements in relationship satisfaction (Cohen’s *d* = 0.69), relationship quality (*d* = 0.57), and relationship confidence (*d* = 0.47), and individual functioning 12 months post-treatment (Doss et al., 2019). The online intervention was significantly less costly than the in-person version of the treatment rendering it appropriate for couples with limited income. The couples who participated in OurRelationship.com reported satisfaction nearly equivalent to that reported after high-quality in-person therapy, supporting the feasibility and promise of couple-based interventions administered through an online modality.

In a recent review of online interventions for couples affected by cancer, Vanstone and Fergus (2020) identified areas of growth in the field over the last decade as well as future potential. Strides have been made, for example, in relation to virtual support programs for intimate dyads coping with prostate cancer. Schover et al. (2012) investigated the efficacy and perceived benefits of an online intervention for heterosexual couples affected by prostate cancer. In a randomized controlled trial, men diagnosed with prostate cancer and their female partners received CAREss sexual counseling face-to-face or through an online modality. Results demonstrated that the online version of CAREss produced equally significant gains in the men’s sexual function and satisfaction as the face-to-face intervention. Two studies of real-time, couple-based interventions delivered via video-conference platforms, one for couples coping with advanced gastrointestinal cancer (Porter et al., 2017), and the other for couples wishing to improve their sexual intimacy after BC (Cullen and Fergus, 2021) showed promise in terms of their feasibility and acceptability to participants. The intervention by Porter et al. (2017), as a pilot randomized controlled trial, also provided preliminary evidence for efficacy in terms of improved relationship satisfaction outcomes based on small to moderate between-group effect sizes for patients (Cohen’s *d* = 0.30) and partners (*d* = 0.34), and between-group effect sizes for patients on communication around affect (*d* = -0.35) and problem-solving (*d* = -0.50) with lower scores signifying improvement.

Adding to the literature on the benefits of remotely delivered couple-based interventions in general, and in relation to cancer specifically, are findings from a randomized controlled trial of the Couplelinks intervention to support younger couples affected by BC (Fergus et al., 2021). The greater burden posed by BC to younger couples, combined with the known benefits of online modalities including greater flexibility, accessibility and convenience (Korp, 2006; Paul et al., 2013; Badr et al., 2015; Kruse et al., 2017) inspired the development of “Couplelinks.” Couplelinks is an asynchronously delivered, professionally facilitated web-based program entailing a series of dyadic exercises and with opportunities for relationship reflection that partners undertake together with the aim of improving their mutual understanding, support, and ability to communicate constructively around the impacts of BC on each partner and the relationship (described further below). In the RCT, couples from across Canada were randomized to treatment or waitlist control conditions. The analysis, based on 31 couples in the treatment group and 36 couples in the waitlist group, showed modest improvements in positive dyadic coping (Cohen’s *d* = 0.24) and perceived ability to cope as a couple with BC (*d* = 0.23), but effects were not maintained at 3-month follow-up and no effect was seen on overall relationship adjustment or satisfaction (Fergus et al., 2021).

### Current Study

The purpose of the present study was to evaluate participant satisfaction with Couplelinks based on their quantitative and qualitative responses to a non-standardized Treatment Satisfaction Questionnaire (TSQ) administered approximately 1–2 weeks after program completion. The primary research objectives were to understand participant perceptions of: (1) the online intervention’s structure and content; (2) the professional facilitation component; and (3) the ways in which the program did and/or did not benefit them.

## Materials and Methods

### Procedures

The study upon which this analysis was based received ethics approval by the following institutions: Sunnybrook Health Sciences Centre and York University in Ontario (ID# 300-209); QEII Health Sciences Centre in Nova Scotia (ID# 2010-357); the British Columbia Cancer Agency (ID# H10-00300); and Cancer Care Manitoba (ID# 2013-017).

The current study is based on an analysis of feedback collected from 53 participants representing 30 couples who completed the Couplelinks program as part of an RCT (Fergus et al., 2021; 23 dyads and seven individual partners). Couples were eligible for participation in the RCT provided that (1) they were in a committed (i.e., married, cohabitating, engaged, or dating for at least 6 months) heterosexual relationship, (2) the female partner was 50 years or younger and had a diagnosis of invasive, non-metastatic breast carcinoma within the previous 36 months, (3) both partners were fluent in English, and (4) they had access to a reliable Internet connection. Couples were excluded from participation if they were currently in couple counseling or if they intended to partake in couple counseling over the course of the study period. Additional exclusion criteria included mental illness that could hinder either partner’s progress through the program such as severe depression, psychotic disorders, or substance abuse. The presence of interpersonal violence or abuse in the relationship also excluded couples from participating in the study. The inclusion/exclusion criteria were assessed over the phone via a detailed screening interview protocol at the time of initial contact with all participating patients and partners individually.

Couples were actively recruited for the RCT by health care providers at collaborating institutions in Ontario, Nova Scotia, Manitoba, and British Columbia. In addition, flyers were displayed in hospitals and cancer agencies, announcements were made during hospital meetings and in BC support groups, and links to the informational webpage of Couplelinks were posted on social media and on other online BC resources. The recruitment and treatment period spanned 5 years from 2010 to 2015. A total of 75 couples were randomized to treatment (*n* = 39) and waitlist control (*n* = 36) groups. Participants were informed of their randomization outcome after baseline measures had been completed. Seven couples dropped out of the treatment arm (three withdrew before beginning the program, and an additional four dropped out during the program). One additional couple was omitted from the analysis as they neglected to read the facilitator’s asynchronously delivered feedback, and thus were considered to not have experienced an integral component of the treatment.

### Couplelinks Online Intervention

#### Conceptual Underpinnings

“Couplelinks” as an online relationship enhancement program for young couples coping with BC, was designed to help partners improve their communication, communal support, self-other knowledge and mutual perspective-taking vis-à-vis the illness and their relationship in general (Fergus et al., 2014, 2015). The intervention, entailing a series of sequentially delivered dyadic exercises (described further below), is rooted conceptually in systemic-constructivist metatheory (Fergus and Reid, 2001; Reid and Ahmad, 2015) which emphasizes couple intersubjectivity and reflexivity (i.e., building upon partners’ implicit understandings of self and other, and their capacity to conjointly reflect upon relationship dynamics so as to improve these). The overarching goal of Couplelinks is to help partners strengthen their mutual bond and sense of “we-ness” in reference to the shared stressor of BC, and to function more effectively as a team (Fergus and Reid, 2001; Fergus, 2015). In a marital therapy context, strengthening we-ness mediated improvements in relationship adjustment (Reid et al., 2006; Ahmad and Reid, 2016). While Couplelinks, as a professionally facilitated but primarily self-guided program, is distinct from couple counseling, the exercises were nonetheless intended to facilitate relationship reflection in a way that is conducive to fostering we-ness and to tackling BC as a collective challenge (Bodenmann, 2005). Assuming a team-based approach to dealing with BC-related stressors has consistently been shown to allow for better adjustment to cancer and reductions in individual psychological distress (see Brandão et al., 2014).

#### Program Description

The first iteration of the program was developed by the authors and pilot tested from 2008 to 2010 (Fergus et al., 2014), and then assessed in the context of a Canada-wide RCT from 2010 to 2015. Its purpose is to enhance relationship functioning, feelings of closeness, support and adjustment to cancer, by guiding couples through six experiential exercises or “Dyadic Learning Modules” (DLMs) that are intended to strengthen the couple’s listening skills, emotional and physical intimacy, and positive affect in the relationship, as well as to promote perspective- taking and the ability to engage in open, constructive communication about cancer (see Supplementary Material 1 for list of DLMs). Each module focuses on a theme fundamental to relationships such as “Creating Connection,” or “Facing Cancer as a Unified Front” and contains a related experiential exercise the couple must complete for homework, such as creating a visual representation of their experience with cancer. Couples in the program also have the option to complete an additional DLM focused on building concrete communication skills should they and their facilitator agree that would be helpful. Upon completion of each module, couples provide feedback and a written reflection on the experience. Couples are given a timeframe of approximately 8-weeks to complete all six modules. In addition to the six modules, there are psychoeducational articles and video clips relevant to young couples and BC. For example, a video clip shows a younger couple speaking about their experiences with BC, exposing Couplelinks participants to another couple in their shoes as a way of reducing feelings of difference and isolation; written materials focused on issues relevant to younger BC patients such as premature menopause or communicating with children about cancer.

#### Professional Facilitation Component

A personal professional facilitator adds a level of individualization to the standard curriculum of the Couplelinks program. The facilitator provides individual support, guidance, and instruction about the program’s aims, principles and strategies used. The facilitators use a private online space on the Couplelinks website called the “Dialogue Room” to communicate asynchronously with their designated couple via text over the course of the program. A notification is sent to the facilitator upon the couple’s completion of each module along with their feedback and reflections. Feedback is then sent by the facilitator *to* the couple and access to the next module is granted. Each couple receives a phone call from their facilitator after the completion of the second and fourth modules, and has the option of scheduling additional phone conversations as needed (for more detailed description of the intervention see Carter et al., 2015; Fergus et al., 2015; Ianakieva et al., 2016).

### Materials

To evaluate the perceived satisfaction of couples with the Couplelinks program, their responses and feedback were collected through the following:

#### Treatment Satisfaction Questionnaire

After completing the intervention, each partner was asked to complete a Treatment Satisfaction Questionnaire (TSQ). The TSQ guides participants to rate their degree of satisfaction with the program overall, the program’s convenience, and the quality of the professional facilitation on a five-point Likert scale. To obtain written, more in-depth understanding of the program’s perceived value, limitations and benefits, open-ended questions were included such as, “What did you like best about the program?” and “What did you like least about the program?” In the final sections of the TSQ, participants used both Likert scales and open-ended questions to provide specific feedback on the value of the psychoeducational articles and videos. For the last item, there is a space to provide “any additional comments” regarding the overall program (see Supplementary Material 2 for the TSQ).

### Data Analysis

#### Quantitative

Descriptive statistics such as means and standard deviations (SD) were used to summarize the quantitative data obtained from the Likert scales on the TSQ. Generalized linear models with a random intercept for couples to adjust for the within couple correlation using a variance component correlation structure were developed. A fixed effect for gender was entered into the model to test for differences in mean outcome scores. Effect sizes for the difference in outcome scores (females-male) were calculated as model estimated value divided by the pooled standard deviation. The data analysis was conducted using SAS/STAT software version 15.2 and the SAS System for Windows version 9.4.

#### Qualitative

For the data obtained from the open-ended questions in the TSQ, Braun and Clarke’s (2006) procedure for thematic analysis was used. The first and second authors (KF and AT) took the lead on the qualitative analysis. First, the data were repeatedly read to gain familiarity with the depth and scope of its content. Second, recurring themes across participant responses pertaining to the research objectives were systematically identified and collated into meaningful categories in an “open-coding” fashion (Glaser and Strauss, 1967). The analysts met regularly to review codes and discuss their interrelationships. As this analysis was more descriptive than interpretative, achieving consensus was fairly straightforward. Rare differences of opinion were resolved through discussion until a consensus had been reached. Patterns across the dataset were derived inductively from the data themselves rather than from preconceptions based on prior research or theory. Third, the categories were sorted and combined to create meaningful overarching themes. Finally, the prescribed themes were reviewed, revised, and organized into a coherent framework. To ensure quality thematic analysis, themes were generated on the basis of a thorough, inclusive, comprehensive and equally weighted view of the entire dataset rather than from a few vivid data extracts (Braun and Clarke, 2006). Themes were identified at a manifest (vs. latent) level rather than looking interpretively beyond what participants had written (Graneheim and Lundman, 2004).

## Results

### Participant Characteristics

Demographics of the participants and couples are summarized in Tables 1, 2, respectively. A total of 53 participants completed the questionnaire (23 patient-caregiver pairs, and seven individual partners (*n* = *3* women and *n* = *4* men) representing 30 couples in total).^1^ The 26 participating women were an average age of 38.9 years old (*SD* = 5.48). There were no age restrictions for the 27 males, although they were on average 40.8 years old (*SD* = 6.35). Eighty-one percent of the couples were married, 15% were cohabitating, and 4% were living apart. On average, couples had been together for 13.8 years (*SD* = 7.46). At the time of participation, most women were receiving follow-up care (54%), although a sizable portion were undergoing active treatment (31%), or had just finished active treatment (8%). Only two women (8%) were recently diagnosed. Forty-six percent of women had Stage I BC, 19% had Stage II, and 35% had Stage III. The majority of participants (83%) were White and most had completed university or college (86%).

**TABLE 1:** Individual Participant Characteristics (*N* = 53 participants).

	Female (*N* = *26*)				Male (*N* = *27*)			
		
	*M*	*SD*	*n*	%	*M*	*SD*	*n*	%
**Age**	38.92	5.48			40.81	6.35		
**Race**								
Caucasian			21	80.77			23	85.19
Asian			3	11.54			1	3.70
Other			2	7.69			3	11.11
**Highest level of education**								
High-school			1	3.85			3	11.11
College			10	38.26			10	37.04
University			12	46.15			13	48.15
Post-graduate			3	11.54			1	3.70
**Age at diagnosis**	37.50	5.40						
**Stage**								
Stage 1			12	46.15				
Stage 2			5	19.23				
Stage 3			9	34.62				
**Treatment period**								
Recently diagnosed			2	7.69				
Active treatment			8	30.77				
Just completing treatment			2	7.69				
Follow-up			14	53.85				

**TABLE 2:** Couple characteristics (*N* = 30).

	*M*	*SD*	*n*	%
**Marital status**				
Dating/Engaged			1	3.85
Common-law			4	15.38
Married			21	80.77
Length of relationship	13.88	7.46		
Length of marriage	10.90	6.91		

### Quantitative Results

In terms of the Likert indices on the TSQ, participants reported a satisfaction rating of 4.3 out of 5 (*SD* = 0.54) on average, and all but two (both males) indicated that that they would recommend the program to a friend in similar circumstances. In terms of the professional facilitation component, the vast majority of participants agreed or strongly agreed that the facilitator role was necessary (*M* = 4.7, *SD* = 0.60), that the level of interaction with the facilitator was sufficient (*M* = 4.4, *SD* = 0.86), and that the facilitator’s feedback was important (*M* = 4.6, *SD* = 0.60). Participants found the program was generally convenient to use (*M* = 4.09, *SD* = 0.81). Male and female participants differed only on the overall satisfaction variable with females’ satisfaction ratings being significantly higher (*p* = 0.01, Table 3), with a medium effect size = 0.57.

**TABLE 3:** Outcomes by gender (*N* = 53).

	Female (*n* = 26)		Male (*n* = 27)			
		
Variable	*Mean*	*SD*	*Mean*	*SD*	*p*-value*	*ES* **
Program satisfaction	4.46	0.51	4.15	0.53	0.01	0.57
Program convenience	4.15	0.83	4.04	0.81	0.45	0.15
Facilitator feedback important	4.58	0.70	4.48	0.70	0.62	0.11
Facilitator amount of interaction sufficient	4.35	0.98	4.37	0.74	0.83	−0.06
Facilitator role necessary	4.58	0.64	4.67	0.55	0.59	−0.13

*Generalized linear model with a random intercept for couples to adjust for within couple correlation.

**Effect size was calculated as the model estimated mean difference divided by the pooled SD.

### Qualitative Results

The qualitative analysis of the TSQ identified 432 meaningful units, which were collated into 30 codes and 14 overarching themes. The themes and corresponding codes were organized by the research objective that it addressed. Six themes emerged from the analysis of participants’ perceptions of the online intervention’s structure and content. Four themes emerged from the analysis of participants’ perceptions of including an online facilitator in the program. Five themes spoke to the experiential gains participants felt they took away from the program (see Table 4). Direct quotes written by participants on the TSQ were identified by their gender and participant ID. Quantitative data in support of each research objective were incorporated into the qualitative descriptions of the results.

**TABLE 4:** Themes and codes from analysis of the Couplelinks treatment satisfaction questionnaire.

Themes	Codes
**Program structure**	
Curriculum	■ Activity-based learning (e.g., exercises) ■ Psychosocial materials (e.g., videos, articles)
Involvement of both partners	■ Inclusion of the male partner ■ Support for the male partner
Time allotted	■ Insufficient time to complete all modules ■ Sufficient time to complete all modules
Self/couple- guided	■ Easy to progress through program ■ Difficult to stay on task
Convenient and flexible	■ Accommodating of each couple’s schedule ■ Flexibility of online education
Desire for in-person contact	■ Face-to-face sessions ■ Privacy concerns with online self-disclosure
**Professional facilitation**	
Humanized the intervention	■ Skilled feedback ■ Participant-facilitator connection
Motivated couples to progress through program	■ Instruction clarification ■ Accountability ■ Encouragement
Facilitated insight into relationship	■ Skilled reflection by facilitator ■ Helped develop novel insight
Offered reassurance	■ Affirmation (“on the right tract”) ■ Validation and confidence
**Experiential gains**	
Opening channels of communication	■ Opportunity for important conversations ■ Communication skill improvement
Carving out time for each other	■ Opportunity to focus on relationship ■ Quality time and fun
A sense of togetherness	■ Couples feel “in this” together ■ Closeness
Gaining insight into the relationship	■ New or different perspective ■ Identified areas of improvement ■ “There really wasn’t anything shockingly new”

### Program Structure

#### Curriculum

Each couple was expected to complete the program’s standard curriculum consisting of six Dyadic Learning Modules (DLMs), while reviewing the psychoeducational articles and videos was left to each participant’s discretion. The majority of the participants’ comments revealed favorable evaluations of the DLMs such as couples repeatedly reporting on the value of the “the variety of activities,” ^(F13)^ or “the role playing” ^(M16)^ involved in some of the modules. One participant commented, “I found something useful in all the exercises” ^(M4)^ and another wrote, “All aspects were very valuable.” ^(F23)^ However, not every couple was able to derive maximal benefit from the modules or see their value. The most illustrative example of individual factors shaping couples’ evaluations was the dichotomous responses regarding the enjoyment of the “Facing Cancer as a Unified Front” module. In this module, each couple was tasked with using their creativity to build a graphic representation of their illness on the webpage. While a number of participants wrote explicitly about the value of this module, couples who did not perceive themselves as “artistic” wrote that it was challenging and unenjoyable. As one female participant wrote, “The exercise where we did the drawing was outside my comfort zone—I do not like that sort of thing.” ^(F16)^

In terms of the written psychoeducational materials, slightly more than half of participants reported to have read these, and of those that read the articles, the majority indicated they valued them. Over half of participants also watched the psychoeducational videos and the majority of these individuals found them to contain valuable information. As one participant wrote:

The videos provided additional guided support with the various phases of the program. No one really understands how you feel, the fears you have or how cancer has affected your relationship until they’ve been through it. I found that I could relate to many aspects of the… videos. ^(F17)^

However, one psychoeducational video was met with constructive feedback by one couple. Reflecting on the value of the videos, one female conveyed how she could not relate to the couple in one video clip:

They [couple in video] seem to have faced an early stage cancer, since they say they were always clear that they would be okay after the treatment. I could not relate with that case because our main concern was always whether I would survive or not. Treatment side effects were always a minor thing compared to the fear of dying and [names of couple in the video] were apparently in a different situation, where the worst part of the journey is associated with the treatment, side effects. ^(F22)^

#### Involvement of Both Partners

The Couplelinks program importantly provides male partners with the opportunity to receive psychosocial support, addressing each couple as a *dyad*, instead of just the single partner alone: “It was the first “cancer” activity that actively involved both of us.” ^(29F)^ Positive responses regarding the inclusion of the male partner in the program suggest that couples perceive psychological support in cancer care to be more readily available to the identified patient, the woman with BC. As one participant stated: “By participating in a program that involved us as protagonists, it highlighted how important our relationship is and how much care and attention we need to give in order to feel happy with each other.” ^(F20)^

#### Time Allotted

Couples were asked to complete the Couplelinks program within approximately 8 weeks by reviewing the psychoeducational materials, and completing the experiential dyadic exercises on weekly basis. Feedback was split among participants regarding the time allotted for completion. Participants reported either needing “more time” ^(M4, F7, F30)^, feeling “pressure” ^(M3, M25)^ or “pressed for time to complete by the deadline” ^(F30)^ (*n* = 13) *or* that the timeframe was “totally doable” ^(F15)^, “appropriate” ^(M28)^, and “reasonable” ^(M19)^ (*n* = 8). One participant wrote, “It was very difficult to make the time to complete the modules within the allocated time” ^(26F)^ which contrasted with another participant who wrote, “I was given more than enough time to complete each module.” ^(12F)^

#### Self/Couple-Guided

The Couplelinks program was designed and structured to function as an asynchronously delivered, self-managed intervention. The ability to decide when to complete the modules was viewed as a benefit for some couples, and a drawback for others. Some participants wrote about the challenge of “finding the time” ^(M8)^ to fit the weekly exercises into their schedules (*n* = 14), while other participants reported that the program’s flexibility allowed for it to be easily incorporated into their week (*n* = 4). As well, while most participants wrote about the benefits of the program’s convenience and of doing the intervention at their “own pace,” ^(F10)^ some wrote about how it was perhaps a little *too* flexible (*n* = 5). For these participants, the lack of structure was a challenge and made it harder to complete the weekly exercises. As one female participant explained:

It was a little too convenient and I found that despite our ability to do it when we had time, that almost gave us an excuse when we were too busy. For me personally, I need something a bit more structured, and we could have done better if my husband and I scheduled the time between us and kept to the schedule. ^(16F)^

Although feedback regarding the Couplelinks program’s design and structure was mostly split between participants, there were also contradictions observed sometimes within a single participant. The same female participant ^(16F)^ continued:

The flexibility. I loved that and hated it too. I’m sort of like a child that needs to be reminded from time to time, and I really appreciated the support from the moderators. Everyone has been very kind and I felt like they were really on my (our) side.

#### Convenient and Flexible

The primary benefit identified regarding the virtual nature of the intervention was its accommodative nature (*n* = 22). Participants appreciated the freedom to complete the program and exercises at home on one’s “own terms.” ^(9M)^ Accessing the intervention online, and at their own pace, was perceived as an advantage over scheduled appointments or “driving into the city.” ^(6F)^ As one participant put it, “We never felt pressured, and found being able to go online at our own leisure was very convenient.” ^(10M)^ For young couples with busy schedules, juggling early careers or childcare, the flexibility also made psychological intervention more feasible, when it otherwise might not have been possible: “The phone call check in’s were scheduled for a time that was best for [husband’s name] and I, which meant evenings due to his work schedule.” ^(11F)^

#### Desire for In-Person Contact

While many participants stated that they benefited from the convenient and accommodative nature of the online program, the most commonly reported limitation was the absence of in-person contact (*n* = 9). Some participants claimed they were simply a “face-to-face kind of person” ^(5F)^ and preferred to communicate with others through in-person contact. One man wrote: “[I did not like] that it was almost entirely online. But my own bias is for more face-to-face (or on the phone).” ^(M29)^ As well, disclosing personal information through an online modality instead of face-to-face was evidently disconcerting for certain participants. One participant felt “awkward” ^(5F)^ talking on the phone to the professional facilitator. Another participant felt “raw” and “expos[ed]” ^(16F)^ revealing personal information through the online modality without the immediate feedback or validation received in in-person counseling.

### Professional Facilitation

Participant feedback revealed that while couples generally appreciated the online, and standard, components of the intervention, they saw particular value in the facilitator’s contributions to their participation in the program and the way that the facilitator tailored their feedback on the modules to the unique couple and their needs:

“Even though having such a program in an online mode has several advantages, especially for introverted people who may feel a bit uncomfortable and for whom it would be difficult to open and share in front of a third party, it is key to have someone following the process.” ^(F22)^

#### Humanized the Intervention

The vast majority of the participants “strongly agreed” or “agreed” that the professional facilitator’s role was necessary and that their feedback was important to their successful utilization of the program. The facilitator allowed participants to feel they “weren’t alone doing some online program” ^(18F)^ and ensured a smooth progression through the generic aspects of the program.

#### Motivated Couples to Progress Through Program

Another primary benefit identified regarding the professional facilitator was that he or she served as a motivator and helped couples “stay on track” ^(M16)^ (*n* = 19). The facilitator importantly pushed the couples to keep pace and meet their deadlines, as one participant wrote, “if we were left on our own without someone there pushing us a little bit, we may not have completed the course.” ^(5M)^ Participants appreciated having an “encouraging, instructive” ^(19M)^ facilitator to help clarify instructions, ensure the exercises were done correctly, who offered guidance when they were “experiencing difficulties” ^(24M)^ and ensured they “got the most out of each activity.” ^(24F)^

#### Facilitated Insight Into Relationship

The facilitator’s “invaluable feedback” ^(11F)^ helped couples make headway in the program (*n* = 16). Many participants appreciated the facilitators’ skillful reflections on their progress through each module considering these to be a “major benefit” ^(17F)^ of the program. The personalized feedback and support “put a voice and thoughts” ^(1M)^ to problems, helped couples “reflect on things,” ^(5F)^ identify areas for improvement, and afforded them new directions to consider. According to one participant, the facilitator helped “to reframe, interpret, elaborate, redirect, [and] refer.”^(21M)^

#### Offered Reassurance

The feedback also importantly served as a source of affirmation and validation, giving couples the confidence to proceed through the program (*n* = 8). One participant wrote, “I often wondered if my responses to some of the questions were clearly understood, [and] his feedback helped reassure me.” ^(4F)^ Another participant wrote, “The feedback was such a great validation to know that we were on the right track and doing well.” ^(11F)^

### Experiential Gains

A positive evaluation of the Couplelinks program ran through the majority of the couples’ feedback. Thematic analysis of responses revealed four experiential gains participants felt they took away from the program: (a) opening channels of communication; (b) carving out time for each other; (c) gaining insight into their relationship; (d) evoking a sense of togetherness, while a few participants felt; (e) “there wasn’t anything shockingly new” in reference to possible gains from the program.

#### Opening Channels of Communication

Many participants reported that the intervention facilitated a sense of openness within their relationship (*n* = 19). The DLMs and experiential exercises facilitated “open” ^(4F)^ (*n* = 10) discussions between partners, “without requiring someone to initiate a “we need to talk” situation” ^(19M)^ which could seem more ominous. The exercises encouraged, and enabled, couples to “share their experiences and emotions openly” ^(7M)^ and presented an “excuse” ^(4F)^ or opportunity for participants to be “more open, and straightforward” ^(22F)^ with their partners. The Couplelinks program guided couples through “difficult dialogue” ^(23F)^ and for one couple, opened “doors that were closed many years ago” ^(22M)^, evoking important conversations that they might not otherwise have had. As one participant commented: it was “helpful to have something to force us to communicate.” ^(23F)^

Some couples reported a distinct change and improvement in their method of communication as a result of the program (*n* = 5). For example, one female participant shared a concrete way in which she and her partner’s communication ameliorated: “I learned there were hidden feelings deep inside of me, even though I was being positive and optimistic. It’s okay to feel down and express how I feel to my husband” ^(27F).^ The program helped couples “enhance” ^(11F)^ their communication skills, and acquire “better” ^(26F)^ or “new ways to communicate,” ^(6M)^, and “got [them] to talk in a different way.” ^(5F)^ One patient summed this up by saying that, “the program provided several tools that we can use in the future in talking about our feelings [and] concerns.” ^(19F)^

The Couplelinks program helped couples recognize the value of bringing effective and open communication into their relationship (*n* = 10), even “as awkward as the communication might be.” ^(21F)^ In one participant’s written feedback, she reflected, “It’s important for us to keep talking to each other and making time for each other” ^(18F)^, and another participant wrote that the program helped him realize that, “Taking time to listen and talk is key to keeping a healthy marriage.^” (1M)^

#### Carving Out Time for Each Other

Participation in the program provided couples with a set time to spend together each week, “like a date,” as one woman ^(19F)^ put it. One participant commented: “It [was] a way to sort of nurture our relationship after a period of not paying attention to our relationship at all… next to no intimacy, no dates -you know- for a long period of time.” ^(19F)^ Many wrote about enjoying “dedicated time” ^(29M)^ with their partner (*n* = 6), and as one participant articulated, “Having a project that was just about the two of us. No kids involved, no work involved… just the two of us.” ^(21F)^ One participant expressed the gratitude she felt to have the opportunity to spend quality time alone with her partner as “there are also issues of body image and sexuality associated with BC.” ^(26F)^

Partaking in the program also allowed for couples to set specific time aside for focusing on their relationship (*n* = 11). Many couples appreciated that Couplelinks granted them the opportunity to “sit down and breathe” ^(25M)^ or take pause “even in busy times” ^(28F)^ to explore or think about their relationship (*n* = 11). As a female participant wrote, “[It was a] good reason to make time to talk about us.” ^(F18)^

A related benefit that was reported described how participation in the program allowed couples the opportunity to pay special attention to the physical aspect of their relationship. The evaluation of the pilot version of Couplelinks revealed that couples wanted a module aimed to help them reconnect physically and re-engage in sexuality after treatment (Fergus et al., 2014). Thus the RCT protocol included a “Getting Physical” DLM (Fergus et al., 2015) and dedicating time for intimacy contributed to this theme as mentioned by this participant:

“I most liked an opportunity for [name of husband] and I to focus on our sexual relationship. It’s something that had been an issue for us before the program and then cancer treatment only made it that much more difficult. The exercise on reconnecting physically was very helpful for us and helped give us a framework to kick-start the physical side of relationship again.” ^(21F)^

#### A Sense of Togetherness

Participating in the Couplelinks program helped couples recognize, as one participant put it, that they are “in this together” ^(16F)^ (*n* = 15). The DLMs the couples completed together, as a team, allowed participants to remember that they are “coping with BC as a couple” ^(13M)^ vs. as individuals. By facilitating the “opportunity to connect,” ^(28M)^ Couplelinks allowed couples to “deepen” ^(F11)^ their relationships, bringing partners closer together (*n* = 7). As one participant wrote: “… [Couplelinks] made us be together, closer to each other in many levels.” ^(22F)^

#### Gaining Insight Into the Relationship

Couples made reference to how completing the program’s exercises and modules enabled them to view their relationship from a “new and different perspective.” ^(M6)^ As one participant wrote about her husband: “The program allowed me to see him in a new light, it made him more human to me.” ^(16F)^ This change of perspective also allowed for the discovery of new realizations about the nature of participants’ relationships. For example, one woman recognized, “My husband and I need to have projects and things to do together, alone without our sons.” ^(22F)^

This enhanced relationship awareness also allowed participants to identify areas in their relationship in need of improvement (*n* = 18). A female participant wrote: “The program has “showed me” to slow down and be more responsive/considerate and patient with him,” ^(16F)^ while her husband learned: “[The program] helped me realize that I have a lot of work to do to make our relationship work. I need to express myself more and I need to be more attentive/aware of what my spouse is going through.” ^(16M)^ Another female participant came to identify, “We need to pay more attention to connecting physically and taking an emotional chance with each other in order to do that.” ^(6F)^

While gaining new insight allowed some participants to identify areas in their relationship in need of improvement, others found affirmation of their mutual bond by, for example, seeing “how strong (our) relationship is.” ^(9M)^ Another participant wrote, “I learned that [wife’s name] and I have a very strong relationship and that I’m proud of how hard we work at it.” ^(15M)^

Another type of learning gained by the couples was reportedly a “great set of tools and strategies to help for managing relationship ‘stuff’ ^(29M)^.” The strategies learned were “useful and practical” ^(25F)^ and, as one participant wrote, “identified ‘problems/topics’ and helped you develop techniques to address them.” ^(17F)^ Some participants made specific reference to acquiring a framework with which to think about and discuss the relationship as one participant noted, “One concept we still talk about is “Turning Toward and Away” from one another.” ^(5M)^

“There really wasn’t anything shockingly new.” ^13F^ While most participants wrote about the many tools, strategies, and insights learned, some participants reported that they did not get “a lot out of it” ^(15F)^ (*n* = 5). As one participant put it, “Except for the physical exercise, the rest of the components didn’t really share anything new for me.” ^(21F)^

The most common reason reported among couples that expressed a lack of novel learning from the program was due to a perception of an already strong relationship. For example, a female participant wrote:

I, personally, didn’t find most of [the DLMs] helpful, but that, I think, is reflective of the fact that we already think about and talk about the things that were prompted in the modules, so there wasn’t anything new, really. ^(21M)^

Some couples who felt they were in particularly high-functioning relationships perceived to have not benefited from the stock components of the program as much as other couples. The same female participant cited above wrote, “My husband and I found that it mostly just reinforced that we have a very good and supportive relationship. But I can see how it would be very valuable for couples who were struggling.” ^(21F)^

## Discussion

The current study examined participant feedback on an online psychological intervention designed to help young couples cope with BC through the use of dyadic coping strategies, psychoeducation, and weekly experiential learning exercises. Participants unanimously saw the benefit in the convenience and flexibility of the program, and appreciated the intervention’s involvement of both partners. Including a professional facilitator in the online intervention was well received among participants as they humanized the intervention, served as motivation to progress through the program, facilitated insight into their relationship, and offered reassurance. The analysis revealed numerous experiential gains that participants felt they took away from the program. Couplelinks helped to: (1) open channels of communication, (2) designate quality time for couples to spend together each week, (3) evoke feelings of unity and togetherness, and (4) inspire new insight in the relationship.

Among young couples affected by BC specifically, the cost effectiveness and flexibility of self-managed interventions provide further value over face-to-face therapy sessions (Hilton et al., 2000; Gould et al., 2006). The Couplelinks program, which targets the couple as a unit specifically, also corroborates past research demonstrating the important effects of psychosocial interventions that focus on improving relationship quality in adjusting to the psychological and physical effects of cancer (Brandão et al., 2014; Ahmad et al., 2017; Kayser et al., 2017). The generally positive evaluations in the current analysis provide evidence that participants perceive self-guided couple-based psychosocial programs to be beneficial and valuable in their experience managing and coping with BC. Below we discuss findings from this study in the context of the Couplelinks RCT outcomes, and how these tie in with literature on the importance of professional online facilitation. We also consider the subset of couples who found the program banal.

### Discrepancies Between Subjective and Objective Indicators of Benefit

It is interesting to consider overall favorable treatment satisfaction findings relative to the RCT outcomes which demonstrated short term improvement in positive dyadic coping but no between group differences in marital adjustment or relationship satisfaction—and given that participants in the current sample represented 30 of the 31 couples randomized to the treatment arm of the RCT (Fergus et al., 2021). Specifically, experiential gains of improved communication, greater closeness, and increased insight into the relationship, should, in theory, be reflected in improvements on standardized instruments such as the Revised Dyadic Adjustment Scale (RDAS). In hindsight, it is possible that the RDAS, as one of the selected primary outcome measures, lacked sensitivity to the change processes being targeted by the intervention. For example, the relationship satisfaction subscale is comprised of items to do with marital conflict and instability—aspects of intimate relationships which were deliberately *not* targeted by Couplelinks with its relationship enhancement, strengths-based (rather than conflict resolution) focus.

Moreover, the Dyadic Coping Inventory, a measure that was evidently more sensitive to the changes provoked by Couplelinks, showed these changes were not maintained at follow-up (Fergus et al., 2021). In this regard, it is important to bear in mind that the TSQ was completed soon after completing the program when any treatment gains would have been most salient for participants. Disparities in outcomes at different data collection time-points raise questions about the situation specificity of programs like Couplelinks that aid couples in enhancing their bond while participating in the intervention but which diminish over time as “everyday life” takes over and couples are less inclined to make their relationship the focus of their attention (one of the main tasks of Couplelinks). A useful analogy here is of a garden that needs tending. The plants and flowers may not be so far gone as to be failing, but the florae are overgrown and unruly, borders are less crisp, and weeds may have begun to proliferate. Thus the benefits of Couplelinks as a relationship enhancement intervention may well be, almost by definition, temporary. This consideration raises intriguing questions around the value of “booster” modules, and more broadly the importance of “relationship tending” in the maintenance of couple bonds.

### Professional Facilitation as Integral to Web-Based Couple Interventions

While the couples’ subjective accounts regarding the structure and content of the program were mixed (e.g., time allotted per module), feedback on their experience receiving the professional facilitator’s individualized support and guidance was consistently viewed as an asset and integral to their satisfaction and successful completion of the program. Past research on self-managed interventions demonstrates the benefits of incorporating a coach or therapist in the program (e.g., Larson et al., 2007), a finding corroborated in the series of investigations of the population-based online intervention, OurRelationship.com (Doss et al., 2013, 2016; Roddy et al., 2016). An early iteration of this program (Doss et al., 2013) illustrates how increasing a program’s scope or mass-influence may limit the impact and effectiveness it can have on single individuals. To maximize the program’s reach, adoption, implementation and maintenance among a nationally representative sample, OurRelationship.com was kept brief and initially did not include professional facilitators. A facilitator was added to later iterations of the intervention and investigators found that, importantly, the professional facilitation enabled couples to gain maximal benefit from the program (Roddy et al., 2016). The presence of a facilitator reduced dropout rates due to increased feelings of accountability to complete each of the program’s activities. This finding, too, is consistent with the current analysis; many couples reported they would not have completed the Couplelinks program without having the facilitator helping them “stay on track” ^(7F)^ and progress through the program.

### Ceiling Effects With Relationship Enhancement Programs

A notable weakness of the Couplelinks curriculum is illustrated by the subset of couples that reported that they did not “learn anything shockingly new.” A commonly reported reason for this was that they felt their relationship was already strong and functional (e.g., “I know for us [Couplelinks] wasn’t overly challenging. But that’s partially because we are just who we are. Like we do openly talk and discuss things frequently. We’re very like-minded.” ^(9F)^) While this can suggest that the Couplelinks program may be less effective for couples who embark on the program with a higher level or quality of relationship functioning, other couples were *both* well-adjusted *and* able to benefit from the program’s content. Indeed, a primary goal of the Couplelinks program was relationship enhancement, and many functional couples did in fact experience novel learning. This disparity may be due to the fact that the subset of couples who perceived themselves to be well-adjusted and did not report achieving insight through the program viewed the stock curriculum as an affirmation of their relationship strengths (e.g., “It definitely seemed that it was just showing us what we already knew about each other” ^(9M)^). It may have been that these couples already perceived themselves to possess the relationship skills taught in the DLM, or that they were unable to incorporate new learning into a relationship they already saw as strong. This finding highlights both the limitations of interventions with pre-determined content designed for mass administration, as well as the added value of professional facilitation which allows for additional tailoring and personalization to couples whose feelings of affirmation prevent them from acquiring new skills and learning.

### Limitations and Future Directions

One limitation of the current analysis is the absence of couple-derived feedback in which partners are addressed together and able to build from one another’s reflections about the program in a co-constructed fashion. Given the intervention is focused on developing “we-ness,” dyadic as well as individual-based evaluations of the program would have been appropriate and likely would have added to the understanding achieved through individual evaluations. An additional consideration is that the sample (*N* = 53) did not include all 62 participants from the RCT treatment arm (*n* = 31 couples). Thus there is the possibility that the present findings were biased toward more favorable impressions of the intervention. Having said that, the fact that there was representation from 30 of the 31 RCT couples in the present sample, with more males than females participating (and it was the male participants who were comparatively less satisfied with the program according to the outcome X gender analysis with this sample), lends confidence to the validity of our findings.

Another sample-related limitation is that this treatment satisfaction analysis was limited to the RCT participants, which was comprised of mainly White couples with post-secondary education inclined to volunteer for novel interventions such as Couplelinks. This result points to a potential self-selection bias or that needing to have Internet and computer access may have posed a barrier to couples of lower socioeconomic status. Moreover, all couples were heterosexual. Future research is needed in order to determine if same sex couples and patients or partners of more diverse ethnicities and educational backgrounds would have the same evaluations. Lastly, the subset of participants who indicated face-to-face counseling would have been preferred should not be overlooked. This finding stands as an indication that traditional counseling should still be available for those who prefer it over a self-directed program, and who have the resources to access it.

## Conclusion

The current study uncovered the perceived benefits and limitations of an online intervention for young couples coping with BC, as well as examined the experience of those using it. The current analysis demonstrates the perceived value of an online, predominantly self-managed, couple-based psychological intervention with personalized support and guidance for young couples with BC. The reported benefits provide support that couples view online interventions to be viable, flexible, accommodative and an unencumbered alternative to traditional face-to-face couple counseling. Findings from the current study support the feasibility and acceptability of the Couplelinks program for couples coping with BC while offering directions for improvement of online couple-based intervention in cancer care.

## Data Availability Statement

The datasets presented in this article are not readily available because participants did not provide consent to share the information in a publicly accessible database. Questions regarding the datasets should be directed to KF, karen.fergus@sunnybrook.ca.

## Ethics Statement

The studies involving human participants were reviewed and approved by the Sunnybrook Health Sciences Centre and York University in Ontario (ID# 300-209); QEII Health Sciences Centre in Nova Scotia (ID# 2010-357); British Columbia Cancer Agency (ID# H10-00300); and Cancer Care Manitoba (ID# 2013-017). The patients/participants provided their written informed consent to participate in this study. Written informed consent was obtained from the individual(s) for the publication of any potentially identifiable images or data included in this article.

## Author Contributions

KF: conceptualization, formal analysis, funding acquisition, investigation, methodology, project administration, resources, supervision, visualization, writing original draft, and review and editing. AT: methodology, data curation, formal analysis, writing original draft, and review and editing. SA: conceptualization, data curation, formal analysis, investigation, methodology, project administration, supervision, visualization, and review and editing. SG: conceptualization, data curation, formal analysis, funding acquisition, methodology, supervision, visualization, writing original draft, and review and editing. EW: conceptualization, funding acquisition, investigation, methodology, resources, and writing–review and editing. DM and JS: conceptualization, funding acquisition, investigation, methodology, resources, and writing-review. WC: conceptualization, investigation, methodology, and writing-review. AP: data curation, investigation, methodology, and project administration. All authors contributed to the article and approved the submitted version.

## Conflict of Interest

The authors declare that the research was conducted in the absence of any commercial or financial relationships that could be construed as a potential conflict of interest.

## Publisher’s Note

All claims expressed in this article are solely those of the authors and do not necessarily represent those of their affiliated organizations, or those of the publisher, the editors and the reviewers. Any product that may be evaluated in this article, or claim that may be made by its manufacturer, is not guaranteed or endorsed by the publisher.

## Footnotes

1 Both partners completed the TSQ independently and in some instances, only one member of the couple completed it. Although efforts were made to encourage non-completers to provide their feedback, these were ultimately not successful and the research team felt it was important to not ask too much of participants who had already given a lot to the project, and who would also be completing a lengthy follow-up test battery at a later date. Moreover, the TSQ was originally administered as a separate online survey but later became integrated with the full post-treatment test battery. Thus once this integration occurred, TSQ completion rates improved. In the case of one couple, neither partner completed the TSQ and thus of the 31 couples comprising the treatment condition for the RCT, 30 are represented in this evaluation.
