# Supplementary material for: Treatment Satisfaction With Couplelinks Online Intervention to Promote Dyadic Coping in Young Couples Affected by Breast Cancer
Source: Front Psychol. 2022 Jun 16;13:862555. doi: 10.3389/fpsyg.2022.862555 (PMC9245623; doi:10.3389/fpsyg.2022.862555)
Supplement: Supplementary file 1 [file Data_Sheet_1.docx]

**Supplementary Materials**

**S1 Couplelinks - Dyadic Learning Modules (DLMs):**

| **DLM** | **Theme** | **Goal** | **Activity** |
| --- | --- | --- | --- |
| 1 | *Celebrating our Strengths* | To create an opportunity for partners to reflect upon and communicate about their individual and shared strengths. Individual strengths consist of qualities in the other that one values, enjoys, or admires. Shared strengths include those that define the couple relationship as strong or resilient and help in the process of coping with BC. | Independently, each partner enters 10 positive qualities about their partner online. Then the partners brainstorm together about the strengths they share as a couple in general. They are then asked to choose from this list the couple strengths that they bring to bear on their experience with BC. The data is transformed into an image of a tree with roots and foliage, with the individual and couple strengths. The couple is asked to review and discuss these together. |
| 2 | *Understand-*  *ing your Partner’s Inner World* | To help partners more accurately understand the other’s thoughts and feelings in relation to BC based on the assumption that previous relationship schemas may have to be revised or altered in the context of the illness. | Independently, each partner answers a series of questions about their own and their partner’s preferences and experiences progressing from trivial to more serious topics (including cancer-related). These lists are then reviewed together in order to stimulate discussion and clarification. |
| 3 | *Creating Connection* | To help partners become more aware of the other person’s ‘bids’ for interaction and support, and to pay attention to their own ‘turning toward’ and ‘turning away’ behaviours on a day-to-day basis (Gottman, 1991). | Over the course of the week, each partner is asked to attend to his or her own turning toward and away behaviours. These are  tracked and recorded online. At week’s end, the couple reviews and discusses their entries that appear in chart format. |
| 4 | *Facing Cancer as a Unified Front* | To assist couple in adopting a team orientation in relation to BC (i.e., a sense of ‘us’ versus ‘it’). Also, to foster the attitude that the illness is a shared experience (i.e., not ‘belonging’ to woman with cancer). | Couple guided through an exercise designed to get them thinking metaphorically about cancer, and then to create a visual representation of the illness in order to fortify sense of ‘we-ness’ in relation to cancer. |
| 5 | *Getting Physical* | To assist couples in reconnecting  physically and sensually as a stepping stone to re-engaging sexually, as many couples find their sexual life is disrupted by treatment. | Independently, each partner considers and records a physically pleasurable shared time in their past. The couple then discusses each memory. Next, couples participate in the Sensate Focus exercise. |
| 6 | *Looking Back and Moving Forward* | To assist couple with moving forward after BC by situating the illness in the context of the larger relationship history and by having the couple consider new goals and directions for themselves (particularly in the wake of lost goals and dreams). | Together partners co-construct a relationship line illustrating pivotal events/periods in their shared history (i.e., high and low points). The online program transforms relationship events and phases inputted by the couple into a relationship line. This forms a basis for discussion. |
| Optional | *Intentional Dialogue* | To learn a communication skill that partners can use to share their concerns more effectively and increase their understanding of the other person’s perspective. | Couple watches instructional video clip of another couple demonstrating an Intentional Dialogue. Couple attempts this on own around neutral and more meaningful topics. Enters completion dates online. |

*Note** Table adapted from previously published table (Fergus et al., 2015)

Carter, W., Fergus, K., Ahmad, S., McLeod, D., & Stephen, J. (2015). Defining the role of the online therapeutic facilitator: Principles and guidelines developed for Couplelinks, an online support program for couples affected by breast cancer. *JMIR Cancer*, *1*(1), Article e4. <https://doi.org/10.2196/cancer.3887>

Fergus, K., Ahmad, S., McLeod, D. L., Stephen, J., Gardner, S., Pereira, A., Warner, E., & Carter, W. (2015). Couplelinks - an online intervention for young women with breast cancer and their male partners: Study protocol for a randomized controlled trial. *BMC Trials*, *16*(1), Article 33. https://doi.org/10.1186/s13063-014-0534-8

Fergus, K., Carter, W., McLeod, D., & Lewin, J. (2011). *Couplelinks.ca: Program Facilitation Manual – Version 1.0.* pp. 1-95. Unpublished Manuscript

Fergus, K. D., McLeod, D., Carter, W., Warner, E., Gardner, S., Granek, L., & Cullen, K. (2014). Development and pilot testing of an online intervention to support young couples’ coping and adjustment to breast cancer. *European Journal of Cancer Care, 23,* 481-492. doi:10.1111/ecc.12162

Gottman, J. M. (1999). *The marriage clinic: A scientifically based marital therapy*. New York: W. W. Norton & Company.

Hendrix, H. (1988). *Getting the love you want: A guide for couples*. New York, NY: Harper and Row.

Ianakieva, I., Fergus, K., Ahmad, S., Pos, A., & Pereira, A. (2016). A Model of Engagement Promotion in a Professionally Facilitated Online Intervention for Couples Affected by Breast Cancer. *Journal of Marital and Family Therapy*, *42*(4), 701-715.

**S2**

**Treatment Satisfaction Questionnaire**

We are asking for your assistance in providing feedback about the Online Couplelinks Program you recently completed.  Your responses will be kept strictly confidential, and your name will not be associated with any of your comments.

**Program Evaluation:**

Overall, how satisfied were you with the Couplelinks program?

       1                             2                          3                        4                      5

     Very                    Dissatisfied          Neither Satisfied              Satisfied                     Very

Dissatisfied               nor Dissatisfied                                   Satisfied

Please elaborate:

______________________________________________________________________________________________________________________________________________________________________________________________________________________________________________________________________________________________________________________________________________________________________________________________________

Overall, I found the program to be convenient:

      1                             2                         3                        4                     5

Strongly                  Disagree                 Neither Agree                  Agree                    Strongly

Disagree    nor Disagree                                        Agree

Please elaborate:

_____________________________________________________________________________________________________________________________________________________________________________________________________________________________________________________________________________________________________________________

What did you like best about the program?

________________________________________________________________________________________________________________________________________________________________________________________________________________________________________________________________________________________________________________________

What did you like least about the program?

________________________________________________________________________________________________________________________________________________________________________________________________________________________________________________________________________________________________________________________What was the most valuable thing you learned?

________________________________________________________________________________________________________________________________________________________________________________________________________________________________________________________________________________________________________________________

Were there any components (e.g., couple exercises or written information) that you did not find informative or helpful?  If so, please specify: ________________________________________________________________________________________________________________________________________________________________________________________________________________________________________________________________________________________________________________________

Are there any ways that we could improve this program?  Please be specific about what you would like to see changed:

________________________________________________________________________________________________________________________________________________________________________________________________________________________________________________________________________________________________________________________

Did you read facilitator responses addressed to both of you in the Dialogue Room?

**□**  Yes       **□** No

The facilitator’s written feedback was important to our progress through the Couplelinks program.

     1              2         3                 4        5

Strongly                   Disagree               Neither Agree             Agree                Strongly

Disagree                                                  nor Disagree                                         Agree

Please elaborate:

__________________________________________________________________________________________________________________________________________________________________________________________________________________________________________

The total amount of interaction (i.e., online and phone) with the facilitator was sufficient.

1              2         3                 4        5

Strongly                   Disagree               Neither Agree             Agree                Strongly

Disagree                                                  nor Disagree                                         Agree

Please elaborate:

__________________________________________________________________________________________________________________________________________________________________________________________________________________________________________

The role of the facilitator is a necessary component of this program.

     1              2         3                 4        5

Strongly                   Disagree               Neither Agree             Agree                Strongly

Disagree                                                  nor Disagree                                         Agree

Comments:

__________________________________________________________________________________________________________________________________________________________________________________________________________________________________________

**Informational Section:**

Did you read any of the articles located under the Living with Breast Cancer tab?

**□**  Yes       **□** No

If yes, overall, how valuable was the information that you received from the articles you read?

     1            2     3            4         5

No value               Little to no              Some                   Quite a bit                 Great deal

 at all                      value                    value                      of value                     of value

Did you watch the Listening Dos and Don’ts video?

**□**  Yes       **□** No

If yes, how valuable was the enacted demonstration for you?

     1            2     3            4         5

No value               Little to no              Some                   Quite a bit                 Great deal

 at all                      value                    value                      of value                     of value

**Josi & Keith Videos:**

Did you watch any of the "Josi & Keith" video clips?

         1                                          2                                             3

      None                                   Some                                     Most/all

**If you watched some or most of the video clips:**

Did they add value to your experience of the Couplelinks program?   **□** Yes **□**  No

Please elaborate:

______________________________________________________________________________

Please indicate the extent to which you liked the “Josie & Keith” video clips

    1                         2                             3                               4                               5

Not at all             Very little        Neither liked nor          Quite a bit                   A lot

                                                        disliked

Would you recommend the Couplelinks program to another couple facing breast cancer?

**□** Yes  **□** No

Please elaborate:

____________________________________________________________________________________________________________________________________________________________

_____________________________________________________________________________

**General:**

Have you ever participated in any other couples counselling or educational programs (e.g., premarital classes)?   Yes  _____ No   ______

If yes, how did this program compare to the one(s) in which you previously participated?

________________________________________________________________________________________________________________________________________________________________________________________________________________________________________________________________________________________________________________________

Any additional comments?

________________________________________________________________________________________________________________________________________________________________________________________________________________________________________________________________________________________________________________________

*Thank you very much for your feedback!*
